# Wet Photolithography From Hydrogen Abstraction of a Quasi‐Orthogonal Aggregation‐Induced Emitter

**DOI:** 10.1002/advs.202408979

**Published:** 2025-01-06

**Authors:** Chen Cao, Huan Chen, Jia‐Ming Jin, Ji‐Hua Tan, Hong‐Ji Tan, Jiu‐Dong Lin, Wen‐Cheng Chen, Yi Yuan, Ze‐Lin Zhu, Chun‐Sing Lee

**Affiliations:** ^1^ Center of Super‐Diamond and Advanced Films (COSDAF) and Department of Chemistry City University of Hong Kong Hong Kong SAR 999077 P. R. China; ^2^ School of Chemical Engineering and Light Industry Guangdong University of Technology Guangzhou 510006 P. R. China; ^3^ WISPO Advanced Materials (Suzhou) Co. Ltd. No. Building 12, 200 Xingpu Rd, SIP Suzhou 215000 P. R. China; ^4^ School of Chemistry and Chemical Engineering University of South China Hengyang 421001 P. R. China

**Keywords:** acridone derivatives, aggregation‐induced emission, conformation, hydrogen atom transfer, isotope effects

## Abstract

A new aggregation‐induced emission (AIE) luminogen is obtained by dimerizing acridin‐9(10H)‐one (Ac), an aggregation‐caused quenching (ACQ) effect monomer via an N─N bond and forming 9H,9′H‐[10,10′‐biacridine]‐9,9′‐dione (DiAc) with D_2d_ symmetry. The quenching of DiAc in solution is ascribed to the enhanced basicity promoting hydrogen bonding and then a hydrogen abstraction (HA) reaction and/or an unallowed transition in frontier orbitals with the same symmetry facilitating intersystem crossing. It is found that emissive Ac is one product of the non‐emissive DiAc solution in the HA reaction activated by UV irradiation. By exploiting the AIE properties and the HA reaction of DiAc, photolithographic patterning is demonstrated with a paper wetted with DiAc solution.

## Graphical abstract

An acridone dimer produced by a straightforward oxidation reaction to achieve aggregation‐induced emission (AIE) property leads to fluorescence conversion of the non‐emissive dimer solution by photo‐induced hydrogen abstraction reaction, which could be applied to wet photolithographic patterning.

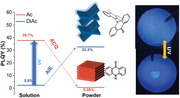

## 1 Introduction

Most organic luminogens tend to be more emissive in dilute solutions but dim in aggregated states due to their typical aggregation‐caused quenching (ACQ) nature.^[^ ^1^ ^]^ In contrast, some luminogens are found to be poorly even not emissive in dilute solutions but show much‐enhanced emissions in aggregated states, which are referred to as aggregation‐induced emissions (AIE).^[^ ^2^ ^]^ Since then, the connotation of AIE has continuously been burgeoning as various AIE‐active molecules were developed and, importantly, their usefulness in analytical and biological applications.^[^ ^3^, ^4^, ^5^ ^]^ Generally, designing luminogens with AIE characteristics (AIEgens) lies in boosting the non‐radiative rate (*k*_nr_) of the molecules to realize *k*_nr_ > *k*_r_ (radiative rate) in dilute solution and restricting/prohibiting the non‐radiation pathways in aggregation. Researchers are showcasing AIE‐active molecules by controlling non‐radiation pathways like vibronic coupling, access to the conical intersection, access to the dark state, and photochemical reaction.^[^ ^5^, ^6^ ^]^ Most new AIEgens are constructed with these AIE‐active molecules as subunits to provide controllable non‐radiation pathways in different states.^[^ ^3^, ^5^ ^]^ Exploring new AIE mechanisms is highly plausible as it opens a new venue to achieve novel and diverse AIE materials and the possibility of innovative applications.^[^ ^4^, ^7^, ^8^ ^]^

Hydrogen abstraction (HA) reaction, an important photochemical reaction contributing to the birth of photochemistry,^[^ ^9^, ^10^ ^]^ now has been developed as a hydrogen atom transfer reaction and found its usefulness in currently active research fields like photocatalyzed aliphatic C─H functionalization, electro‐catalyzed water oxidation.^[^ ^10^, ^11^, ^12^, ^13^ ^]^ Conventional ketone‐based catalysts are prone to form pinacol dimers and have relatively low reactivity, which limits their usage in edge‐cutting research.^[^ ^10^, ^11^ ^]^ These ketones are metal‐free and cheap, fitting the need for green chemistry and large‐scale production. If a novel design can tackle the side reaction and enhance reactivity, ketone‐based catalysts may find their chance in photo‐/electro‐catalyst applications.

Herein, we report an alternative method to obtain AIE properties by dimerizing an ACQ molecule, acridin‐9(10*H*)‐one (Ac), through an N─N linkage to form *9H,9′H*‐[10,10′‐biacridine]−9,9′‐dione (DiAc). Ac is a planar aromatic ketone showing weak emission (photoluminescence quantum yield, PLQY = 0.38%, vide infra) as powder and strongly solvent‐dependent PLQYs (1.5% in cyclohexane and 97% in ethanol).^[^ ^14^, ^15^, ^16^ ^]^ Early studies found this dramatic change is related to the accessibility to ^1^(π,π*) → ^3^(n,π*) intersystem crossing (ISC) process and quenching at the triplet state.^[^ ^14^, ^17^ ^]^ Another series of studies revealed the quenching of aromatic ketone is associated with the hydrogen‐bonded complex between the solvent and solvate.^[^ ^18^, ^19^, ^20^, ^21^, ^22^ ^]^ The hydrogen bonding is promoted by reasonable substituents and can act as an accepting mode to deactivate the excited state or facilitate electron/proton‐transferring HA reaction.^[^ ^18^, ^19^, ^20^, ^21^ ^]^ In either case, dimerization of Ac into DiAc enhanced the quenching rate by facilitating the ISC process or promoting the formation of hydrogen bonds (vide infra).

## 2 Results and Discussion

**Figure 1:**
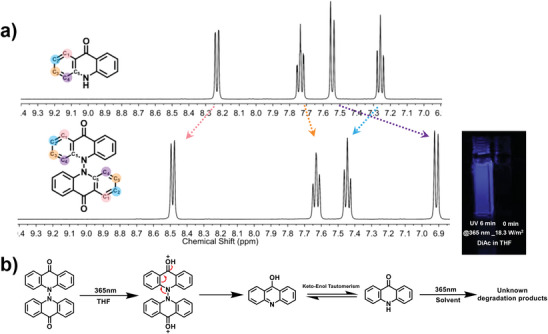
a) The comparison of ^1^H‐NMR spectra collected in DMSO‐*d*_6_ at 400 MHz, the color of the arrows corresponding to the balls, b) The HA reaction of DiAc, inset: the DiAc dilute THF solution under 365 nm at 0 min and 6 min.

DiAc was obtained through the dehydrogenation reaction of Ac monomer by potassium dichromate in acetic acid at 100 °C (Scheme S1, Supporting Information), and synthetic details are provided in Supporting Information (SI). The final compound is non‐emissive in common organic solvents (EtOH, THF, DMSO, *etc*). DiAc can be purified with these solvents and stably stored in ambient atmosphere for over a year. The good thermal stability of DiAc is shown in Figure S1 (Supporting Information). The chemical structure of DiAc was characterized using ^1^H‐, ^13^C‐NMR spectroscopy, high‐resolution mass spectrometer (Figures S2–S5, Supporting Information), and X‐ray single crystal diffraction (vide infra). The *D_2d_* symmetry of DiAc in solution may simplify the ^1^H‐NMR spectrum to compare the chemical shift of Ac and DiAc (**Figure** **1**a; Table S1, Supporting Information), protons in C3‐ and C4‐position are shifted to the higher‐field region and those in C1‐ and C2‐position are shifted to the lower field, indicating electronic density distribution in DiAc is more polarized than that in Ac. Considering protons in the C1‐/C4‐position are closer to an electron pull‐push center (C═O and N atom) with more obvious shifting, the inductive effect should be the dominant factor. Thus, N atoms in DiAc are more electron‐donating, and the basicity of O atoms in carbonyl should also be enhanced, promoting hydrogen bonding between O atoms and solvent molecules. This information found in ^1^H‐NMR spectra is supported by the HA reaction observed in a non‐emissive dilute THF solution of DiAc upon UV irradiation (inset of Figure 1; Supporting Video S1, Supporting Information). Intensive blue emission can be observed from the dark solution after 2 min of UV irradiation (365 nm, 18.3 W m^−2^, Figure S6, Supporting Information). According to the observation and ^1^H‐NMR data, a reaction mechanism in Figure 1b is proposed: first, the non‐emissive excited DiAc will abstract two hydrogen atoms from THF (the solvent itself or the trace amount moisture in it) leading to electronic rearrangement and eventually breaking of the N─N link between two the Ac units. Next, the intermediate will undergo keto‐enol tautomerism and form a more stable keto‐isomer (acridone, with two aromatic sextets) according to Clar's rule.^[^ ^23^ ^]^

**Figure 2:**
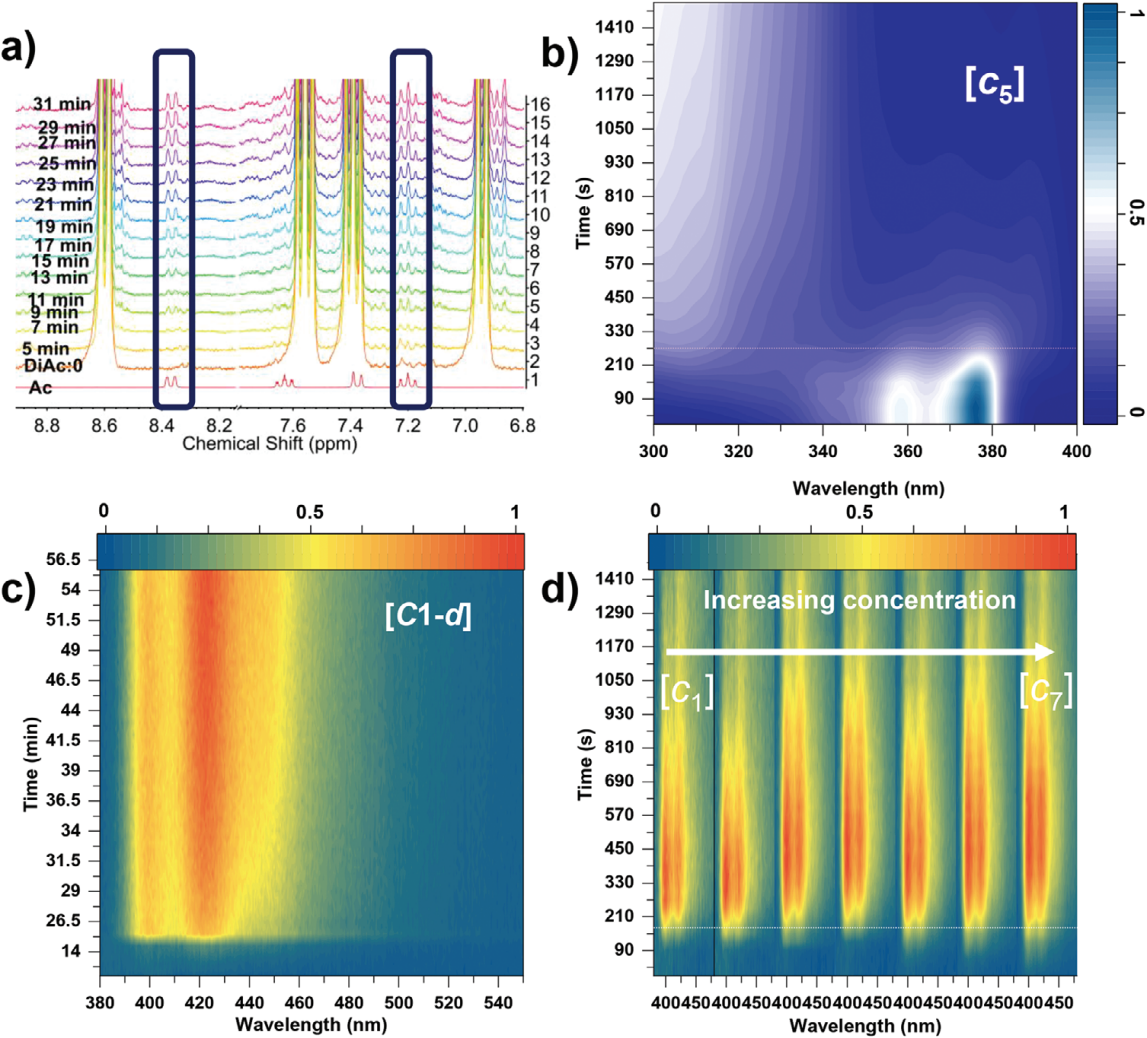
a) The NMR variations in THF‐*d_8_* upon the UV irradiation, b) The TRABS map of [*c_5_*] concentration. c) The TRPL map of [*c_1‐d_*] concentration, d) The TRPL map from [*c_1_*] to [*c_7_*] concentration.

Irradiation time‐dependent ^1^H‐NMR data in THF‐*d_8_* were recorded to verify the proposal. As shown in **Figures** **2**a and S7 (Supporting Information), the acridone signals (peaks in the two rectangle frames) become distinguishable after 7 min irradiation and get more obvious with longer irradiation time. To characterize the existence of HA reaction upon photophysical properties of DiAc solution, we first recorded concentration‐dependent UV‐vis absorption and PL spectra (Figure S8, Supporting Information) of Ac in THF to determine the maximum concentration (c_max_ < 50 µM) that is free from the influence of ACQ in Ac. Next, time‐resolved absorption (TRABS) spectra and time‐resolved PL (TRPL) spectra were recorded in THF solutions of DiAc with concentration [*c*] ≤ 1/2[*c*_max_] (Table S2, Supporting Information). TRABS spectra of DiAc is shown in Figure 2b. Absorption intensities of its 376 nm band drop with UV irradiation time (*T*) and the *T* for the absorption intensity decreasing to half of its original one is ca. 4.5 min. An absorption band at 390 nm can be detected from the UV‐treated DiAc solution, indicating the consumption of DiAc and the generation of Ac. Unfortunately, the large overlap of absorption between DiAc and Ac and other unknown byproducts (high‐energy absorption band, Figure S9a and S10, Supporting Information) deprives further detailed kinetic analysis. TRPL spectra (Figure 2c,d) reveals the isotope effect on this HA reaction, where longer *T* is needed to reach the half of the maximum PL intensity from undeuterated to deuterated solvent ([*c_1_*] in THF/THF‐*d_8_* ≈3/20 min, λ_ex_ = 365 nm). The shorter *T* was obtained by TRPL compared to the one in TRABS (3 min vs 4.5 min) because of the contribution of absorption overlap between Ac and DiAc (Figure 2b,c). *T* remains no change from [*c_1_*] to [*c_7_*] (Figure 2d; Figure S11, Supporting Information), suggesting that the hydrogen donor (i.e., THF) determines the reaction process. This is confirmed with a longer *T* ([*c_1_*], 30 min) observed in the more polar DMSO solvent (weaker hydrogen donor) and a series of parallel experiments in DMSO/DMSO‐*d*_6_ solutions (Figures S12 and S13, Supporting Information). Figure S9b (Supporting Information) shows the PL spectra of Ac and DiAc in THF after UV irradiation. The slight difference in PL profiles is ascribed to the uncertainty of concentration of resulting Ac in the UV irradiated DiAc solution; the ratio of hyperfine peak intensities is correlated to the concentration cf. Figure S8c (Supporting Information). We believe the PL conversion of the DiAc solution is more reliable in reflecting the kinetics of this HA reaction as the dim PL of the DiAc solution. The mass spectroscopy results depicted in Figure S14 (Supporting Information) reveal several important observations. Before UV irradiation (Figure S14b, Supporting Information), the [m+1]^+^ peak of DiAc is observed at 389.5. Additionally, a new peak at 196.1 can be attributed to the resulting monomer Ac following electron spray ionization (ESI). Following 1 minute of UV irradiation (Figure S14c, Supporting Information), the DiAc peak rapidly disappears, while new peaks at 338.5 and 268.5 emerge. Importantly, during this process, no blue emission is observed, indicating the appearance of a non‐emissive intermediate. However, due to the inherent complexity of the ESI process, we are unable to definitively assign the structure of this intermediate. After 4 mins of irradiation (Figure S14d, Supporting Information), the appearance of the acridone can be detected. Notably, the THF solution exhibits a distinct deep blue emission during this stage.

**Figure 3:**
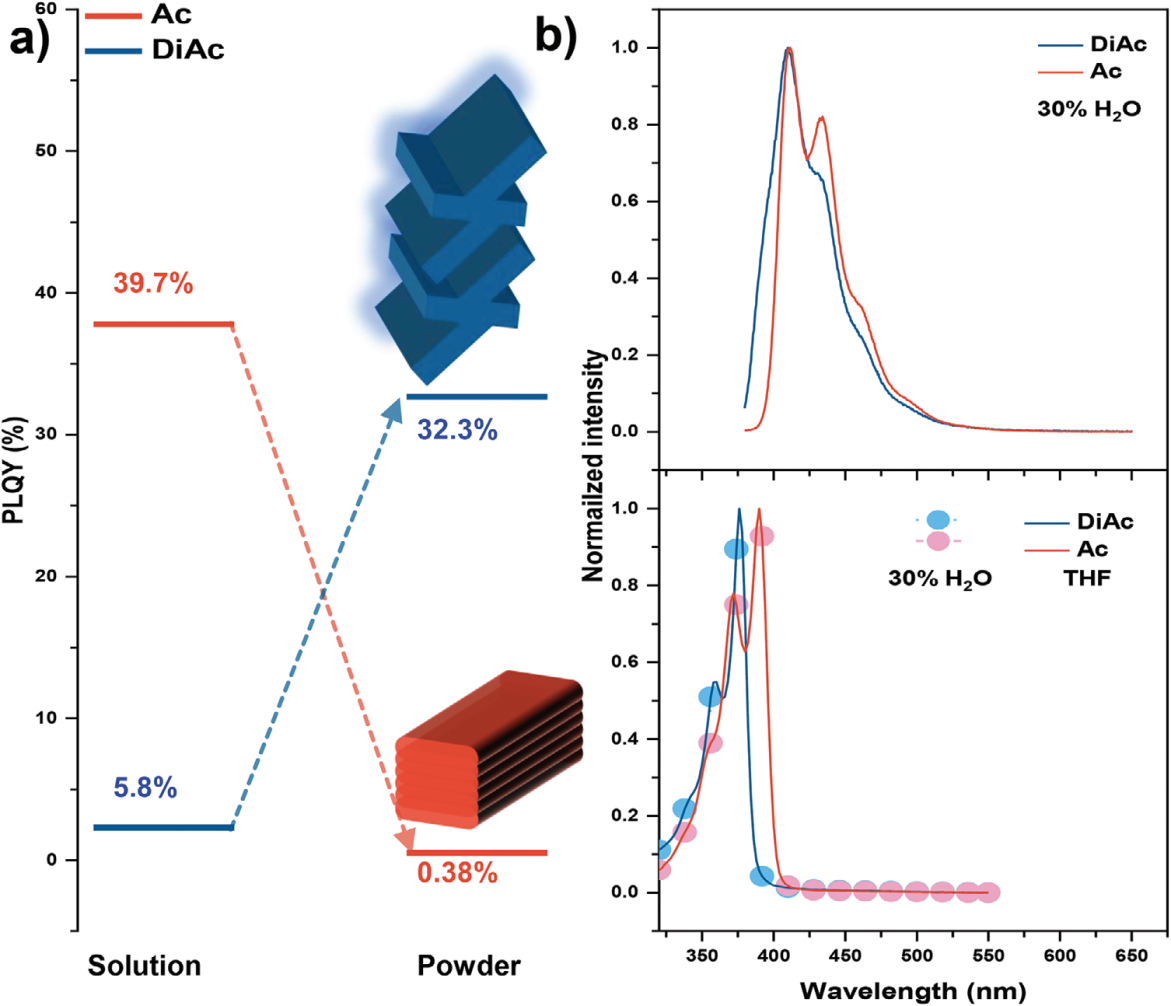
a) The variations of PLQY of Ac and DiAc from solution to powder, b) Upper: the PL spectra of Ac and DiAc in THF with 30% water, and lower: the UV‐vis absorption spectra of DiAc and Ac.

To better understand the quenching mechanism of DiAc in an isolated state, density functional theory (DFT) calculations were performed using the Gaussian 16 program. Dimerizing Ac leads to a *D_2d_* symmetry of DiAc, resulting in a degeneracy and the same orbital symmetry of its HOMO (highest occupied molecular orbital)/HOMO‐1 and LUMO (lowest unoccupied molecular orbital)/LUMO+1 (Figure S15, Supporting Information). Resembling the case in naphthalene,^[^ ^24^ ^]^ the unallowed transition between the frontier molecular orbitals with the same symmetry decreases *k_r_* of the molecule, which facilitates other competing quenching processes like ISC or bimolecular quenching (H‐bonding then HA reaction) in excited DiAc. Time‐dependent DFT (TD‐DFT) calculation further supports the discussion as a zero‐oscillator strength found in the transition of the lowest excited state of DiAc (Table S3, Supporting Information). Consistent with ^1^H‐NMR data, the enhancement of basicity in S_1_ of DiAc is predicted with an increased bond length of C═O from 1.2311 in the optimized S_0_ to 1.3028Å in the optimized S_1_ structures (Table S4, Supporting Information).^[^ ^25^ ^]^ At this stage, it is hard to tell the relative contributions of ISC and the bimolecular quenching. It is intriguing to note that the vibronic hyperfine emission Ac and DiAc are almost identical in the THF/H_2_O system (**Figure** **3**b, AIE property, vide infra), and their photophysical data are summarized in **Table** **1**. The hyperfine absorption bands at ≈350–420 nm can be attributed to locally excited (LE) transitions from the Ac moieties (Figure 3b). The absorption and fluorescence spectra obey the mirror image rule. According to the calculation, the blue shift of absorption of DiAc can be explained by the enlarged energy gap (Figure S16).

**Table 1:** Summary of the photophysical data of Ac and DiAc.

Compound	*E*_g_ ^a)^	HOMO/LUMO^b)^	λ_PL_ ^c)^	λ_abs_ ^c)^	*Φ*_PL_ ^d)^
	[eV]	[eV]	[nm]	[nm]	[%]
Ac	3.06	−6.10/−1.64	410, 433, 462, 495	390, 372	0.38
DiAc	3.16	−6.54/−1.96	410, 433, 464, 496	376, 360	32.3

^a)^ optical energy gap estimated from the absorption onset in THF;

^b)^ calculated at pbe1pbe/def2svp level;

^c)^ Measured in THF solution with 30% fraction of water;

^d)^ Measured in powder.

**Figure 4:**
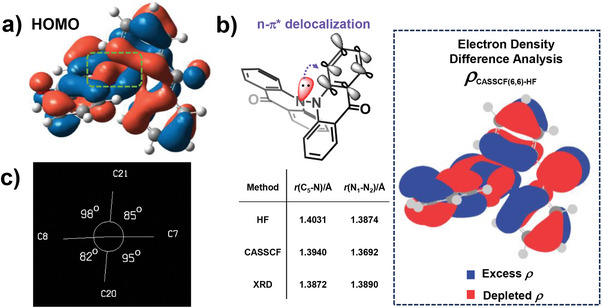
a) The HOMO distribution of DiAc, in green dash line: the N─N entanglement of HOMO, b) Top: the scheme of n‐π* delocalization in DiAc. Bottom left: effect of n‐π* delocalization on key geometric features of DiAc. Right:[CASSCF(6,6)]–[RHF] electron density difference analysis plot, c) The Newman projection plot based on the sight down on the N─N axis.

In aggregation, the bimolecular quenching by solvent molecules is wiped out and the interactions between DiAc molecules are greatly reduced, benefiting from their near orthogonal configuration. As shown by X‐ray diffraction (XRD) of the single crystal, the correct symmetry of DiAc in aggregation would be in the *D_2_* point group, as shown in **Figure** **4**c, due to the crossover angle of 82° along with 3° bending of one Ac plane, which hampers intensive π‐π interaction with a large slippery distance over 3.2Å and 3.8Å between centroids (CCDC: 2 338 999, Figures S17 and S18, Supporting Information). It is interesting to find that DiAc's N─N distances fall to 1.389 Å on average, which is notably shorter than the N─N single bond length in hydrazine(≈1.449 Å).^[^ ^26^ ^]^ The HOMO distribution of the DiAc showing the entanglement shape around the two N atoms (Figure 4a), which hints a certain “n‐π* delocalization” from the lone‐pair electron (LPE) of one N atom to π* orbital of the opposite Ac unit. The double n‐π* delocalization shortened the N─N bond length in DiAc as the enhanced pull strain (n‐π* delocalization, compared to hydrazine) can balance the Pauli repulsion of nitrogen atoms induced by LPE (push strain) in a shorter distance. To better describe the effects of the delocalization interaction in DiAc, we employed ab initio calculation with a (6,6) active space for providing an improved description of the correlation of the electrons in the N─N bond by including the static correlation (Figure. S19, Supporting Information). Figure 4b shows an electron density difference analysis comparing the Hartree‐Fock and CASSCF (6,6) densities. n‐π* delocalization presented as charge depleting from the p orbital of the nitrogen (red lobes) and delocalizing onto the π* orbital of the opposite Ac plane (blue lobes). The n‐π* delocalization is also believed to facilitate the electronic rearrangement in formation of enol tautomerism (Figure 1b), providing another support to the proposed degrading mechanism.

**Figure 5:**
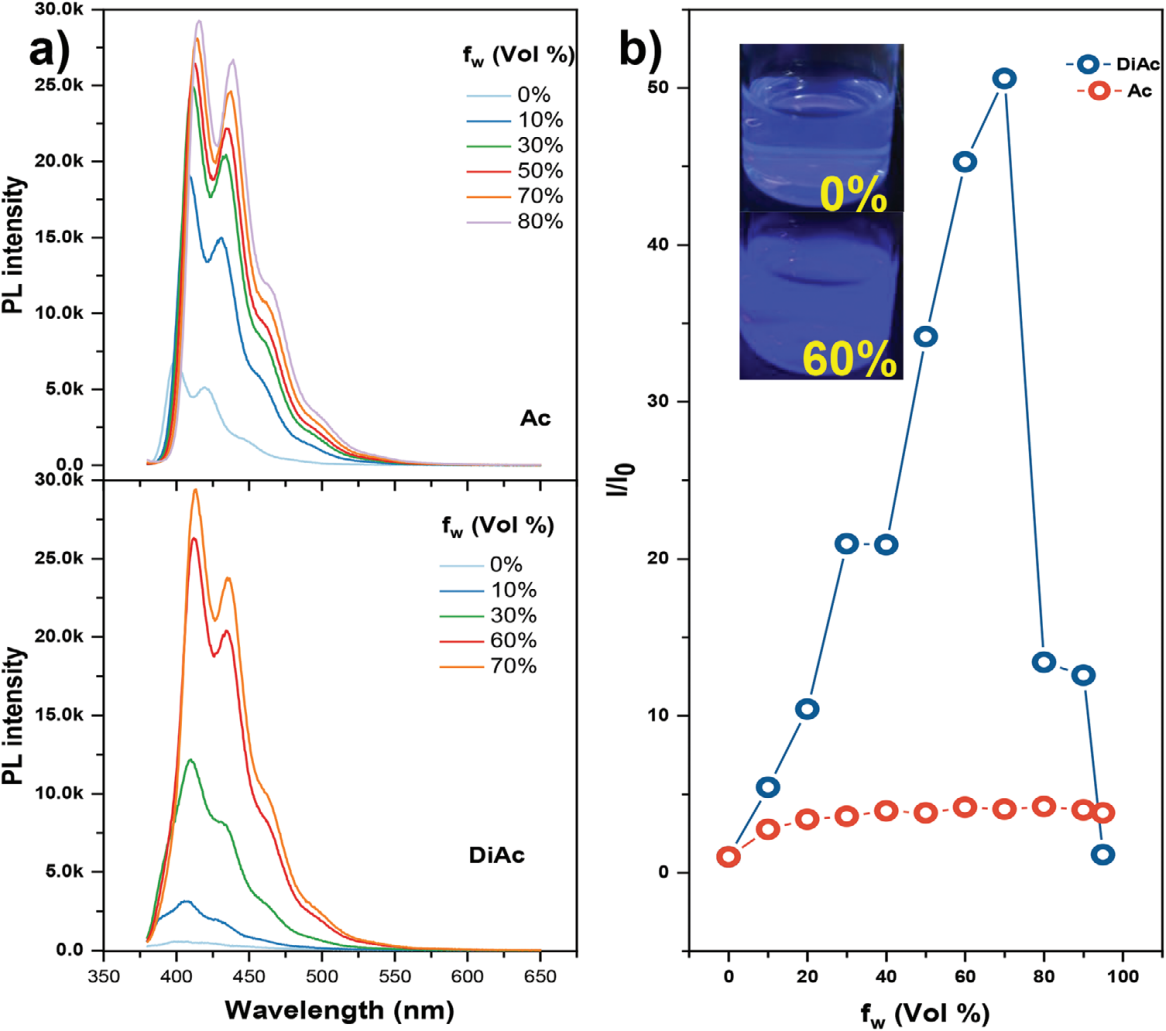
a) The PL spectra of Ac and DiAc in THF/H_2_O mixtures with different water fractions (λ_ex_ = 365 nm), b) The plot of relative emission intensity (I/I_0_) at 410 nm versus the composition of the THF/H_2_O mixtures, incest: photo of DiAc solution under 365 nm UV lamp.

**Figure 6:**
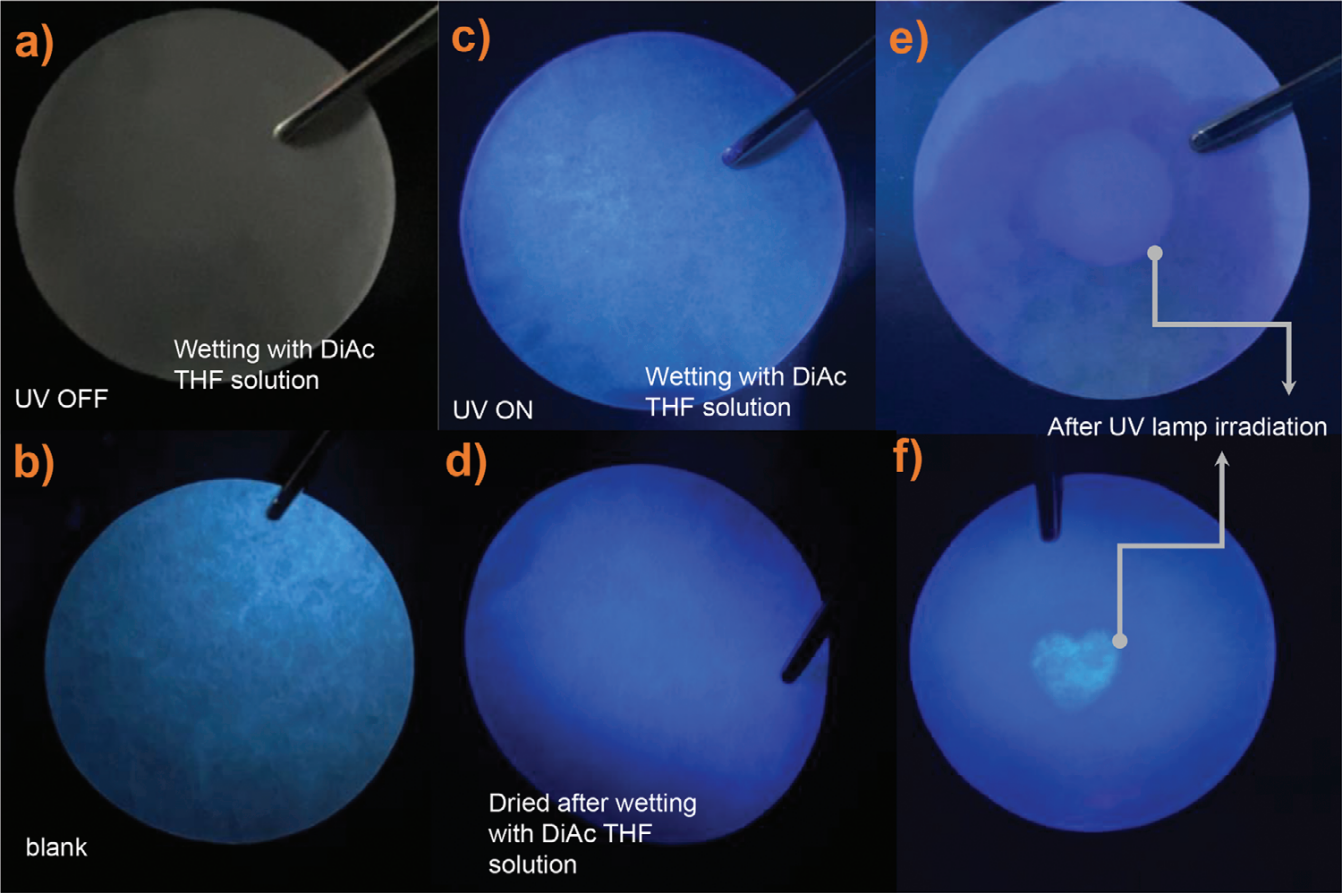
a) The paper wetting in DiAc THF solution at ambient light, b) the dry blank paper under 365 nm light, c) The paper wetting in DiAc THF solution under 365 nm UV light, d) The dried paper after wetting by DiAc THF solution under 365 nm UV light, e) The wetting paper at 365 nm UV light after region UV irradiation(circle shape), f) The wetting paper under 365 nm UV light after region UV irradiation(heart shape).

Next, the AIE property of DiAc is characterized in the THF/water system (**Figure** **5**). Up to 50‐fold PL intensity upsurge is observed in a water ratio (f_w_) of 70%. The PL intensity and UV‐vis absorption decrease with further increase of water ratio due to the reduced solubility of DiAc in the system (f_w_ >70%, Figure S20 and S21, Supporting Information). It is noteworthy that the emission profile of Ac rises as the amount of water increases (Figure 5b; Figure S20c, Supporting Information). This cannot indicate that the Ac would have the AIE character, because the powder of Ac displays typical ACQ (Figure 3a). The PLQY of Ac and DiAc in powder state were determined to be 0.38% and 32.3%, respectively (Figure S22, Supporting Information). The hydrated clusters or proximity effect may clarify the anomalous phenomenon of Ac's emission in water including system.^[^ ^27^, ^28^, ^29^ ^]^ The PLQY of 34.7% and 28.0% for Ac and DiAc with 60% waters are showing in Figure S21 (Supporting Information), respectively. The *k*_r_ and *k*_nr_ are calculated for Ac and DiAc based on the decay profiles of the solutions, as illustrated in Figure S23 (Supporting Information). For Ac, the *k*_r_ and *k*_nr_ values are 2.61 × 10^−2^ ns^−1^ and 4.91 × 10^−2^ ns^−1^, respectively. Similarly, for DiAc, the *k*_r_ and *k*_nr_ values are 2.36 × 10^−2^ ns^−1^ and 6.07 × 10^−2^ ns^−1^, respectively. The AIE property of DiAc should endow it with promising potential for various analytical and biological applications like the other AIEgens. Alternatively, by combining the HA reactivity and its photo‐response in HA reactions, we here demonstrate using a dark solution of DiAc for photolithographic patterning. As presented in **Figure** **6** and Supporting Video S2 (Supporting Information), the paper wetted by DiAc solution is non‐luminous (blue emission to appear at the edges of the paper is caused by the capillary effect of the fibers aggregating DiAc). AIE effect illuminates the dry paper, and blue PL is seen in the dry paper containing DiAc compounds (Figure 6d). Also, the non‐luminous wet paper can be luminated with HA reaction by localizing UV irradiation to enable photolithographic patterning (Figure 6e,f).

## 3 Conclusion

In conclusion, we innovatively construct an AIE molecule (DiAc) with ACQ blocks (Ac), and DiAc shows a ca. 50‐fold PL intensity upsurge in the THF/water system. The non‐emissive property of DiAc solution also helps to discover HA reaction forming emissive products upon UV irradiation. The innate *D_2d_* symmetry and enhanced basicity of the C═O group facilitating HA reaction are possible mechanisms in quenching excitons in solution. These interesting phenomena observed in DiAc enable photolithographic patterning on the paper. This work shows a new working mechanism of AIE molecules, which may significantly diversify AIE molecules by enriching the general design concept and extending the potential application area of AIE‐active materials. Besides, the optical response and enhanced reactivity of DiAc provide new clues to understand and monitor HA reaction and guide the designation of ketone reagents for potential application in photo‐/electro‐catalyzed aliphatic C─H functionalization and water oxidation.

## Conflict of Interest

The authors declare no conflict of interest.

## Supporting information



Supporting Information

Supplemental Video 1

Supplemental Video 2

## Data Availability

The data that support the findings of this study are available from the corresponding author upon reasonable request.
