# Supplementary material for: Wet Photolithography From Hydrogen Abstraction of a Quasi‐Orthogonal Aggregation‐Induced Emitter
Source: Adv Sci (Weinh). 2025 Jan 6;12(8):2408979. doi: 10.1002/advs.202408979 (PMC11848600; doi:10.1002/advs.202408979)
Supplement: Supplementary file 1 — Supporting Information [file ADVS-12-2408979-s003.pdf]

ADVANCED SCIENCE
Open Access

Supporting Information

for *Adv. Sci.*, DOI 10.1002/advs.202408979

Wet Photolithography From Hydrogen Abstraction of a Quasi-Orthogonal Aggregation-Induced Emitter

Chen Cao, Huan Chen, Jia-Ming Jin, Ji-Hua Tan, Hong-Ji Tan, Jiu-Dong Lin, Wen-Cheng Chen, Yi Yuan, Ze-Lin Zhu* and Chun-Sing Lee**

Supporting Information

Wet Photolithography from Hydrogen Abstraction of a Quasi-Orthogonal Aggregation-Induced Emitter

Chen Cao,[a] Huan Chen,[a] Jia-Ming Jin,[b] Ji-Hua Tan,[a] Hong-Ji Tan,[a] Jiu-Dong Lin,[c] Wen-Cheng Chen,[b] Yi Yuan,[d] Ze-Lin Zhu,*[a] Chun-Sing Lee*[a]*

[a] Center of Super-Diamond and Advanced Films (COSDAF) and Department of Chemistry, City University of Hong Kong, Hong Kong SAR, 999077, P. R. China.

[b] School of Chemical Engineering and Light Industry, Guangdong University of Technology, Guangzhou,510006, P. R. China

[c] WISPO Advanced Materials (Suzhou) Co., Ltd. No. Building 12, 200 Xingpu Rd, SIP, Suzhou, P. R. China.

[d] School of Chemistry and Chemical Engineering, University of South China, Hengyang 421001, P. R. China.

General information：

^{1}H and ^{13}C NMR spectra were recorded on a Bruker 400 Hz or 300 Hz NMR spectrometer. All ^{1}H NMR spectra were referenced to $SiMe_4$ through residual ^{1}H resonance(s) of the employed solvent: $(CD_3)_2SO$ (2.50 ppm), THF-d_8(1.72 and 3.58 ppm), ^{13}C NMR spectra were referenced relative to $SiMe_4$ through the resonance(s) of the employed solvent CD_2Cl_2 (54.0 ppm). UV-vis absorption and photoluminescence (PL) spectra were obtained using a HORIBA-Duetta instrument. Absolute PL quantum yield (Φ_{PL}) was measured using an integrating sphere in Edinburgh FLS 980 Fluorescence Spectrometers. The mass spectra were carried out using the Bruker autoflex maX MALDI-TOF/TOF machine and SCIEX API-3200. The molecular geometrical properties were optimized at the pbe1pbe/def2svp level using the Gaussian 16 program package. NTO analysis was carried out by Multiwfn 3.7 package code. HF and CASSCF methods were carried out at def2-tzvpp level. All calculations were carried out under the tolerances of 1.0×10^{-8} Hartrees convergence criteria for the SCF energy change. The single crystals were obtained from the cooling down process of the hot saturated DMSO solutions. The H-bond, dihedral angles, and π-π stacking in crystal data were checked by the Platon program. Aggregation-Induced Emission measurement: Acridone (1.89 mg) or DiAc (4.98 mg) was dissolved in 16 mL of tetrahydrofuran to obtain a ~ 500 μM solution, then 100 μL of the solution was pipetted into 11 sample bottles. The bottles were dried with a heat gun and 5 mL of tetrahydrofuran/water was added to obtain a ~ 10 μM (acridone : 12.1 μM, DiAc: 16.0 μM) solution of different water fractions. Before measurement, the solutions were in ultrasonic shaking for 30min. Then 2 mL of the solution was pipetted into cuvettes for measurement. The standard plot of Acridone measurement: Acridone (8.25 mg) was dissolved in 20 mL of tetrahydrofuran to obtain a 2.113 mM solution, then 50 μL, 100 μL, 150 μL and 200 μL etc. of the solution was pipetted into sample bottles. The bottles were dried with a heat gun and different volumes of tetrahydrofuran was added to obtain a solution range of 11.7389-232.430 μM. Before measurement, the solutions were in ultrasonic shaking for 3min. Then taking 2 ml solution to test UV-vis absorption and PL. The Molar Absorption Coefficient of Ac($\varepsilon = 3.839\times10^{-8}$ Mol^{-1} cm^{-1}) could be obtained by Beer–Lambert law. Reaction kinetics measurement: DiAc (1.65 mg) was dissolved in 15 mL of tetrahydrofuran to obtain a 283.1942 μM solution, then 50 μL, 100 μL, 150 μL, 200 μL, 250 μL and 300 μL etc. of the solution was pipetted into three

sample bottles. The bottles were dried with a heat gun and 3 mL of tetrahydrofuran was added to obtain a range of DiAc solutions (from 4.71990 μM to 28.3194 μM). Before UV treatment, the solutions were in ultrasonic shaking for 3 min. Then taking 2 ml solution to test UV-vis absorption and PL. The UV irradiation experiment was carried out by Analytikjena US 8-Watt lamp(the radiation is 18.3 W/m^2 detected by Macam spectroradiometer), except the patterning experiment was carried out in 5-watt lamp (the radiation is 652.2 W/m^2 detected by Macam spectroradiometer). The supporting video and photos were recorded by the iPhone 11. 4 folds sped up Video 1, and Video 2 was at its original speed.

Thermogravimetric analysis (TGA) was measured on a PerkinElmer STA6000 with a heating rate of 10 °C min^{-1} under a nitrogen atmosphere with a 20 ml/min flow.

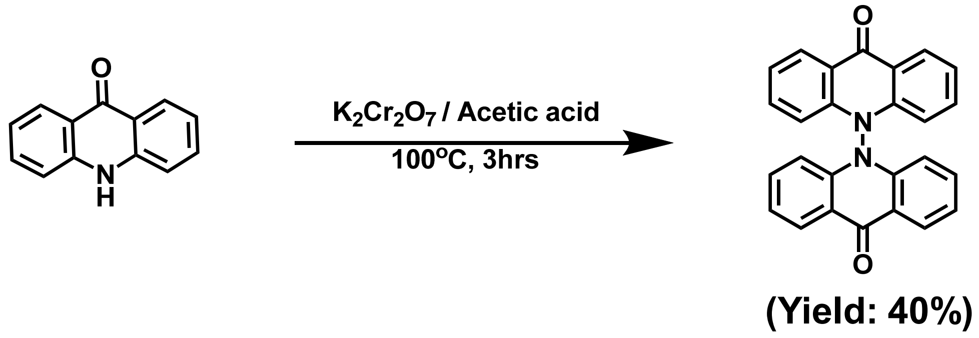

Scheme S1. Synthetic routes of DiAc.

General synthesis:

Reagents and solvents were purchased from BLD pharm, STREM and Dukson or Sigma-Aldrich. Chemicals were used without further purification. 3g (1 eq) of Acridone and 4.53g (1 eq) of $K_2Cr_2O_7$ putting in a single-necked round-bottomed flask and adding 80 ml ice acetic acid in 100 °C reaction. After 3 hours, the reaction solution would become green. Cool to room temperature and add 200 ml of deionized water to generate solids precipitation. After filtration, the precipitate was washed three times with hot water, twice with EtOH, once with DMSO, then with water, and once more with EtOH. Further purified by column chromatography to obtain pure white powder, yield: 40%.

^{1}H NMR (400 MHz, DMSO-d_6) δ 8.49 (d, J = 8.5 Hz, 4H), 7.63 (t, J = 7.6 Hz, 4H), 7.44 (t, J = 7.6 Hz, 4H), 6.91 (d, J = 8.5 Hz, 4H).

^{13}C NMR (101 MHz, CD_2Cl_2) δ 177.64, 141.44, 135.55, 128.65, 124.09, 123.20, 114.24.

m/z calcd for DiAc: 388.12118; found: 389.18317.

Single crystals data: CCDC NO. : 2338999, Temperature: 228(2) K, Wavelength: CuKα (λ= 1.54178 Å), Crystal system: monoclinic, Space group: C_2/c, Unit cell dimensions: a = 9.3165(2) Å / α = 90°, b = 14.7839(3) Å / β = 99.0280(10)°, c = 27.6265(6) Å / γ = 90°, Volume: 3757.98(14) Å^3, Z: 8, Density (calculated): 1.373 g/cm^3, Absorption coefficient: 0.703 mm^{-1}, F(000): 1616, Goodness-of-fit on F^2: 1.054.

The NMR data of Acridone: ^{1}H NMR (400 MHz, DMSO-d_6) δ 11.75 (s, 1H), 8.23 (dd, J = 8.1, 1.5 Hz, 2H), 7.73 (ddd, J = 8.4, 6.9, 1.6 Hz, 2H), 7.54 (dd, J = 8.4, 1.0 Hz, 2H), 7.25 (ddd, J = 8.0, 6.9, 1.1 Hz, 2H).

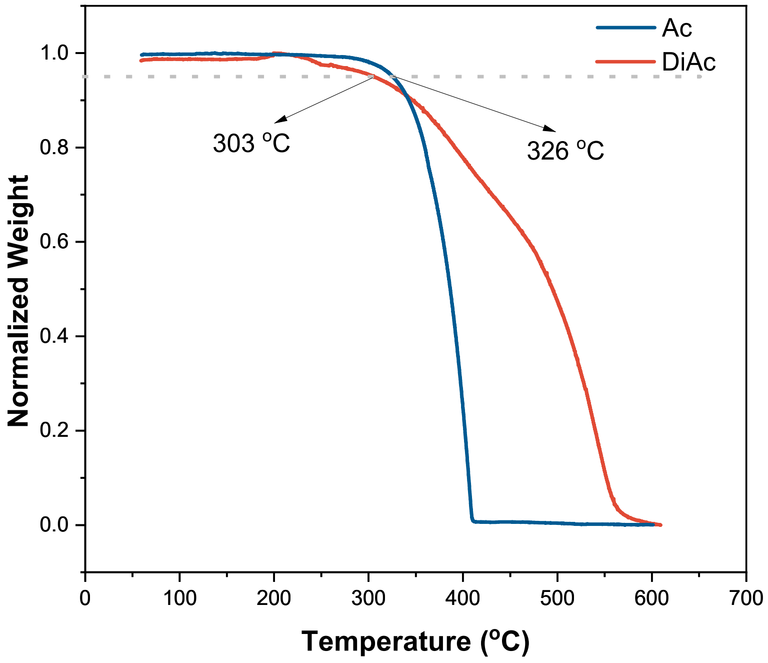

Figure S1. The thermal stability of the DiAc and Ac compounds.

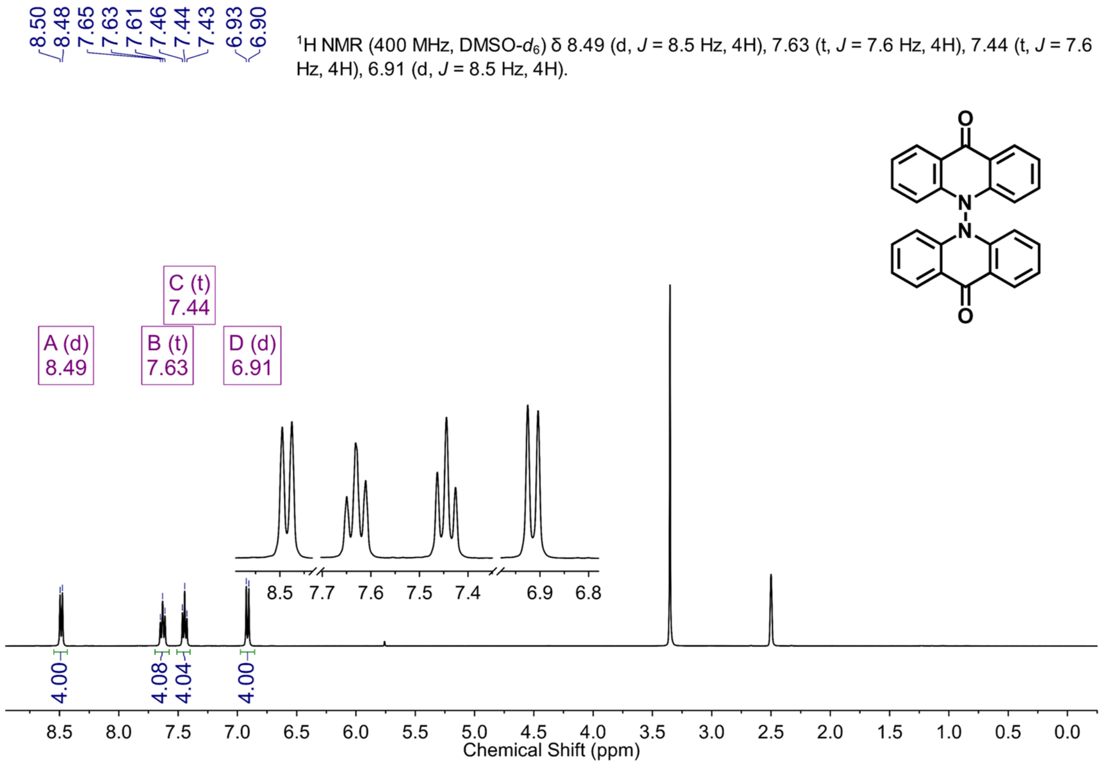

Figure S2. The ^{1}H-NMR spectrum of DiAc.

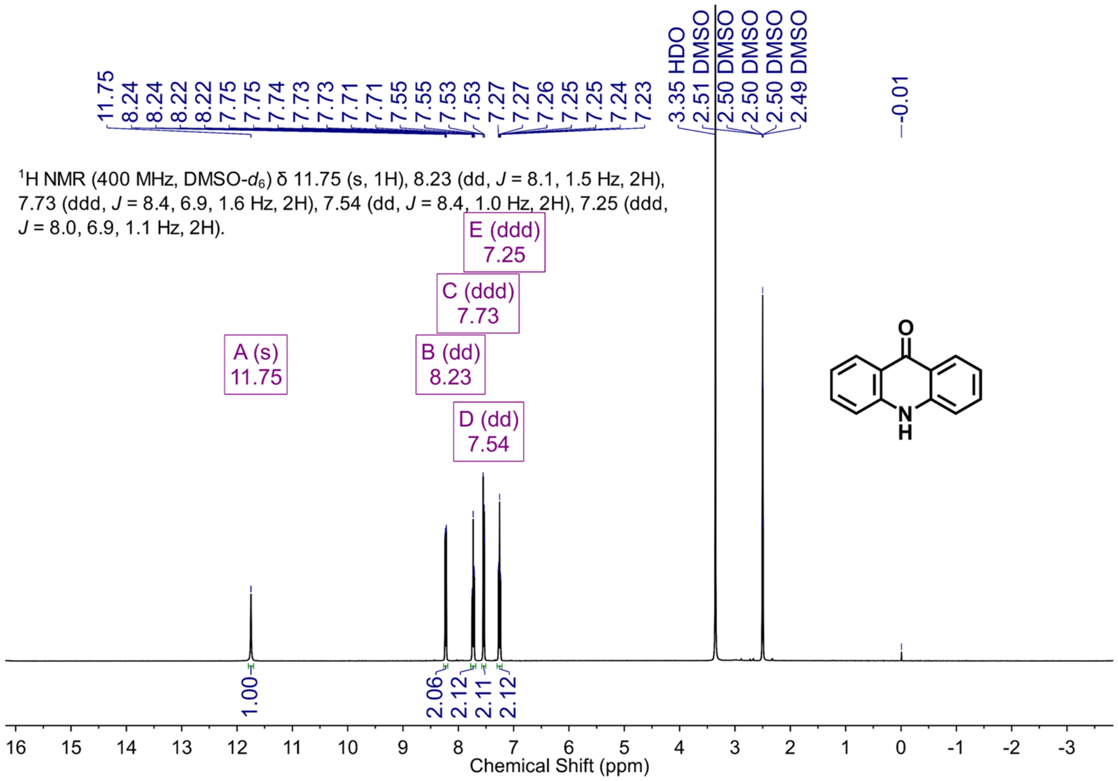

Figure S3. The ^{1}H-NMR spectrum of Ac.

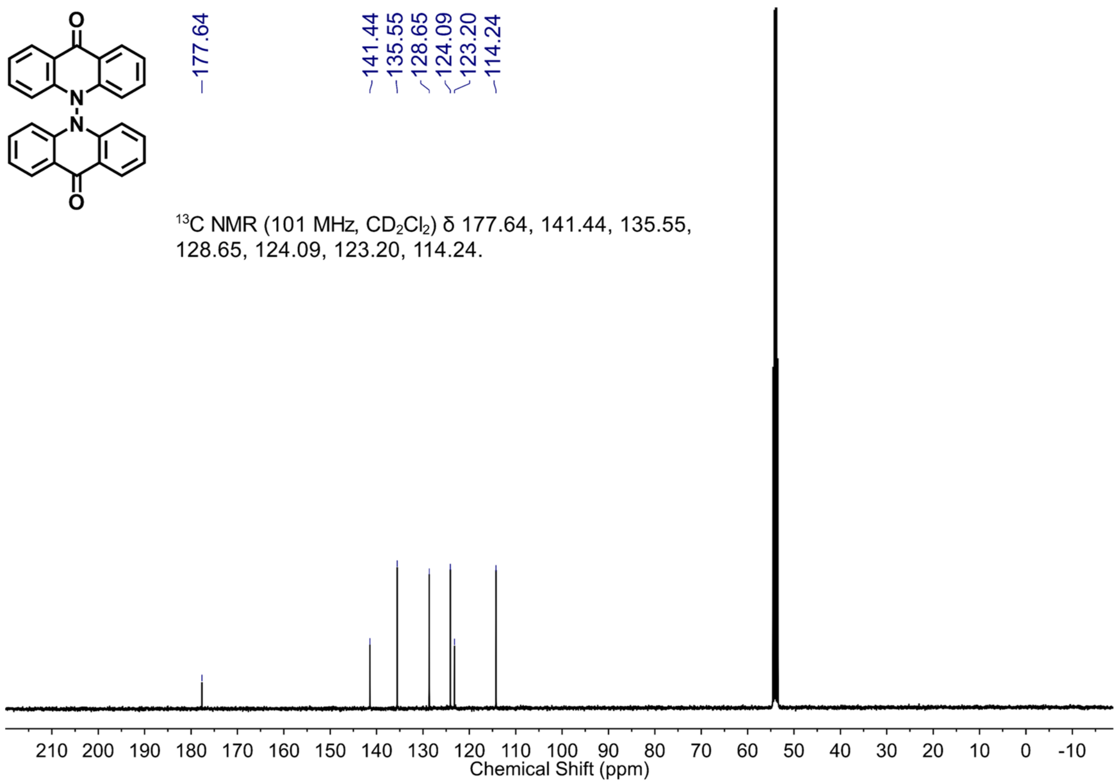

Figure S4.The ^{13}C-NMR spectrum of DiAc.

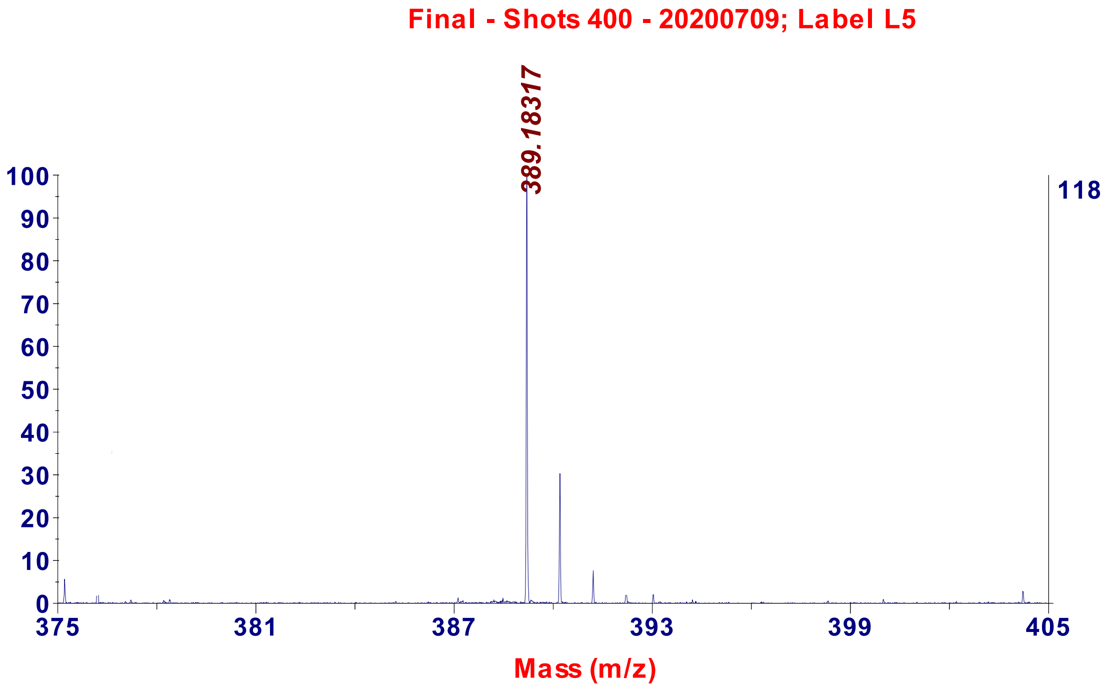

Figure S5. The mass spectrum of DiAc.

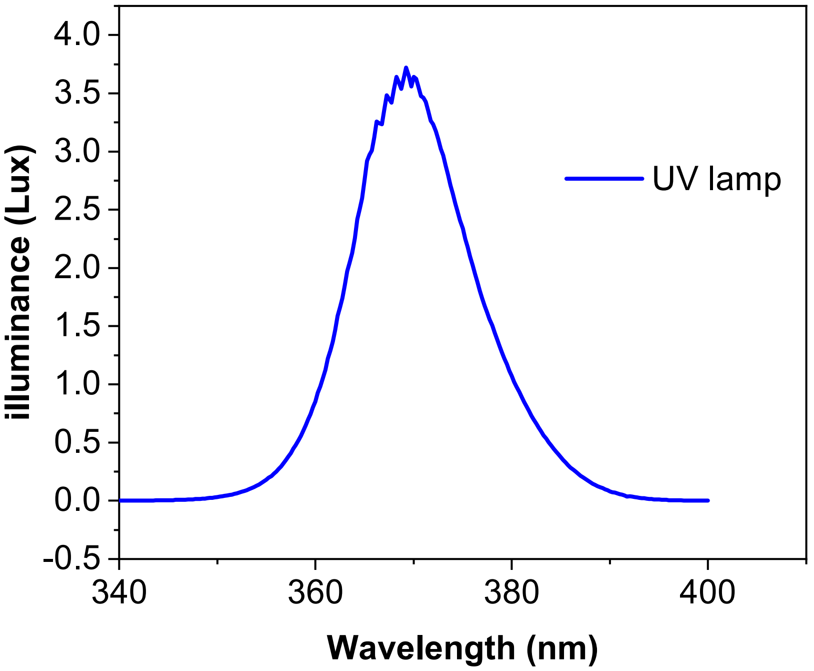

Figure S6. The spectrum of Analytikjena US 8-Watt lamp detected by Macam spectroradiometer.

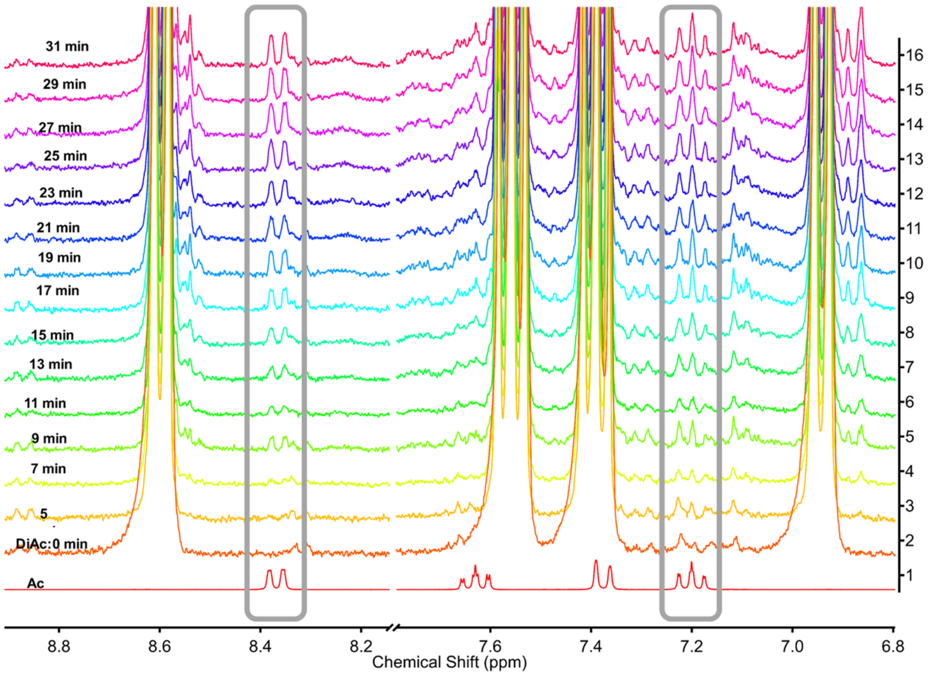

Figure S7. The NMR variation of DiAc in THF-d_8 upon the UV irradiation.

Table S1. The chemical shift of Ac and DiAc in DMSO-d_6

Proton position	δ (central, ppm)		$\Delta\delta$ (ppm)
	acridone	DiAc	
C_1	8.23	8.49	0.26
C_2	7.25	7.44	0.19
C_3	7.73	7.63	-0.10
C_4	7.54	6.91	-0.63

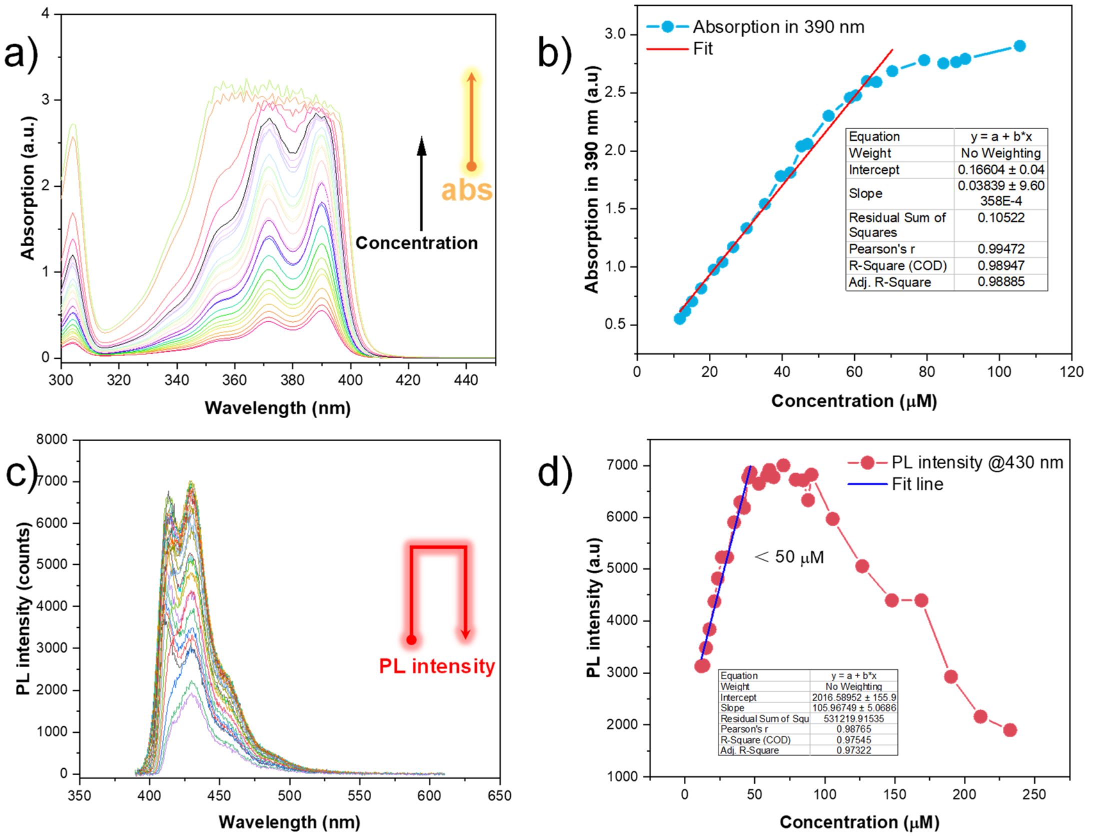

Figure S8. a) The absorption spectra of Ac in different concentrations, b) The absorption versus concentration, c) The PL intensity of Ac in different concentrations, and d) The linear region fitting of PL intensity versus concentration.

Table S2. The concentration detail of DiAc in THF/THF-d_8

DiAc (283.1942 μM)	THF (mL)	End concentration (μM)	Label
100 μL	3	9.43980	$[c_1]$
200 μL	5	11.3278	$[c_2]$
150 μL	3	14.1597	$[c_3]$
200 μL	3	18.8796	$[c_4]$
150 μL	2	21.2396	$[c_5]$
250 μL	3	23.5995	$[c_6]$
300 μL	3	28.3194	$[c_7]$
	THF-d_8		Lable
100	3	9.43980	$[c_{1\text{-}d}]$

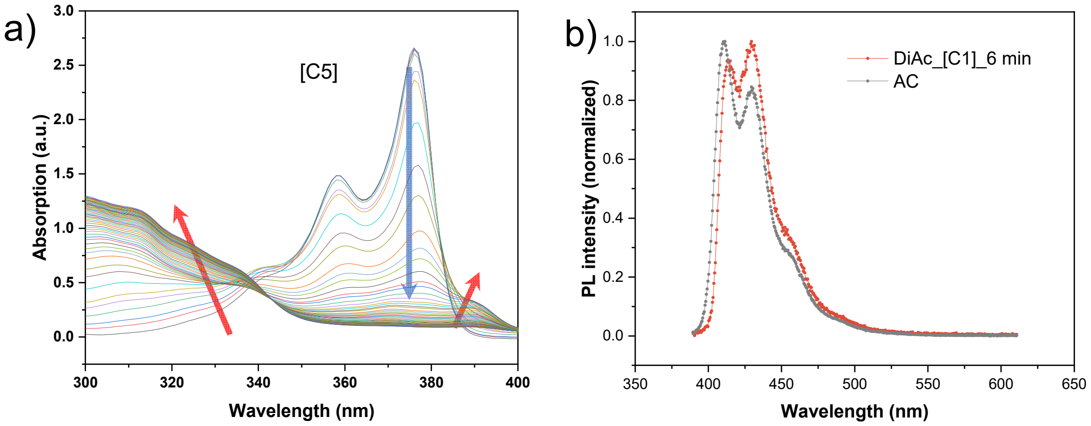

Figure S9. a) The UV-vis absorption variation in $[c_5]$ concentration under different irradiation times, b) The PL spectra of Ac and DiAc after UV irradiation.

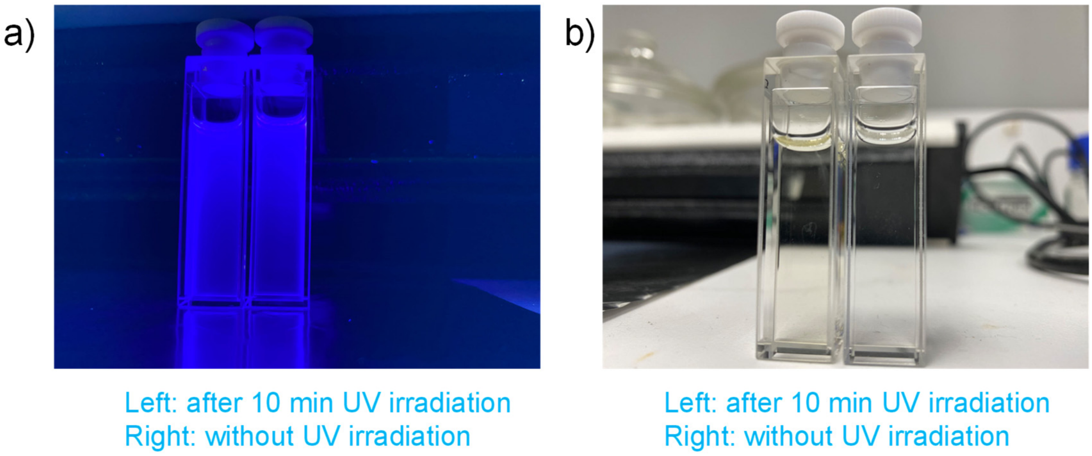

Figure S10. The photography of acridone solution is a) under UV and b) in ambient light.

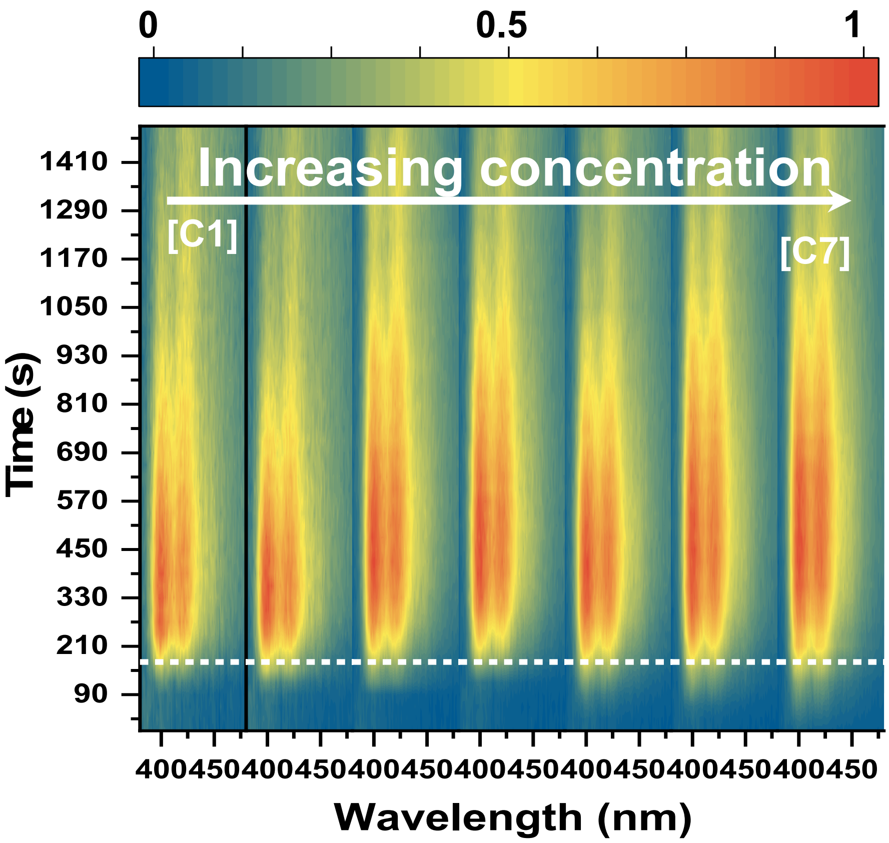

Figure S11. The TRPL map of DiAc with different concentrations in THF.

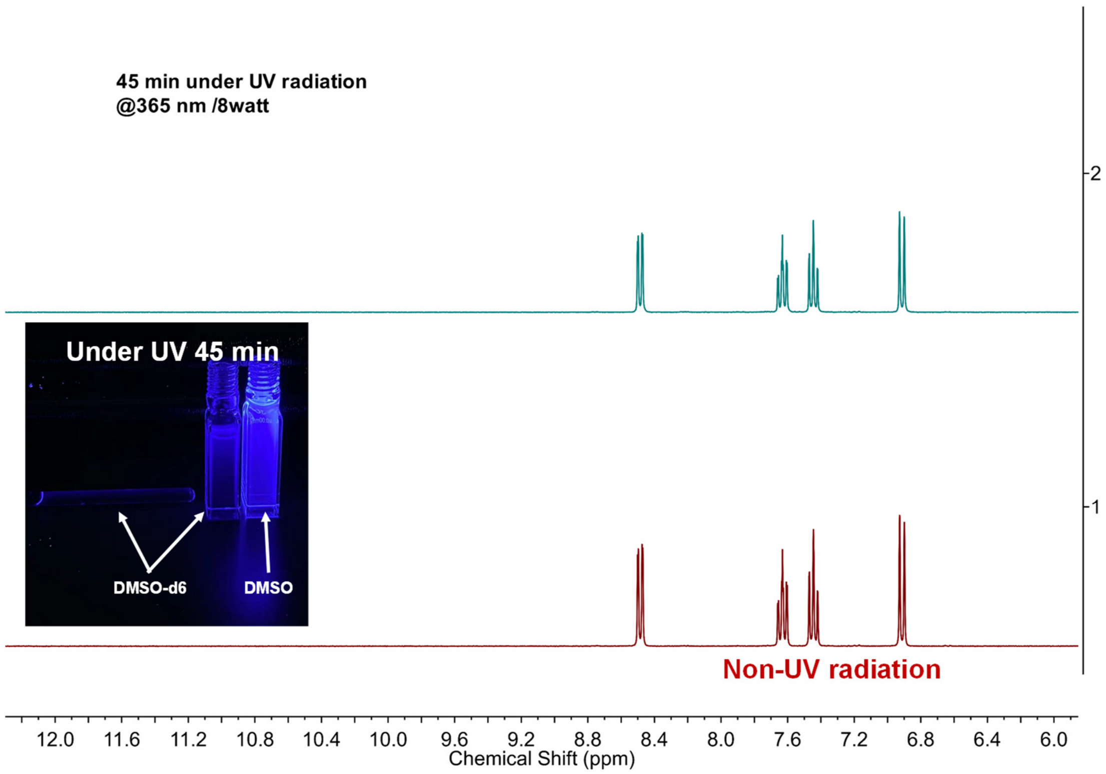

Figure S12. The NMR spectra of DiAc in DMSO-d_6 with different UV irradiation times.

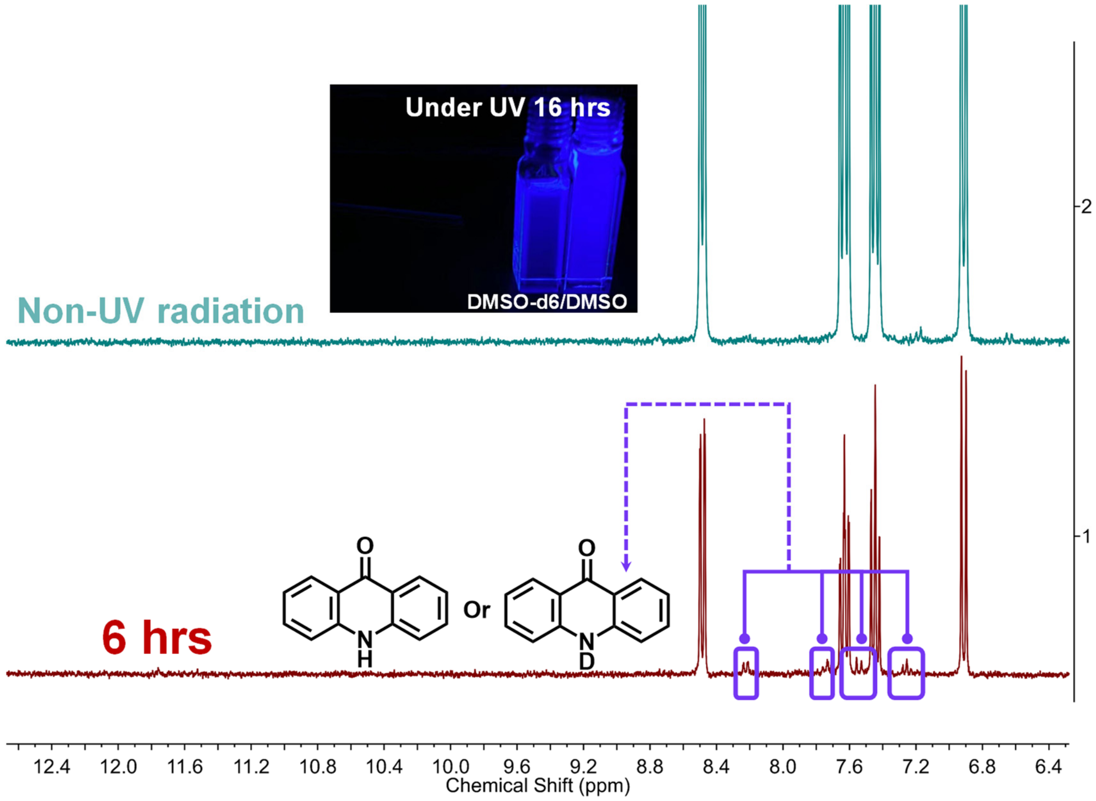

Figure S13. The NMR spectra of DiAc in DMSO-d_6 with different UV irradiation times.

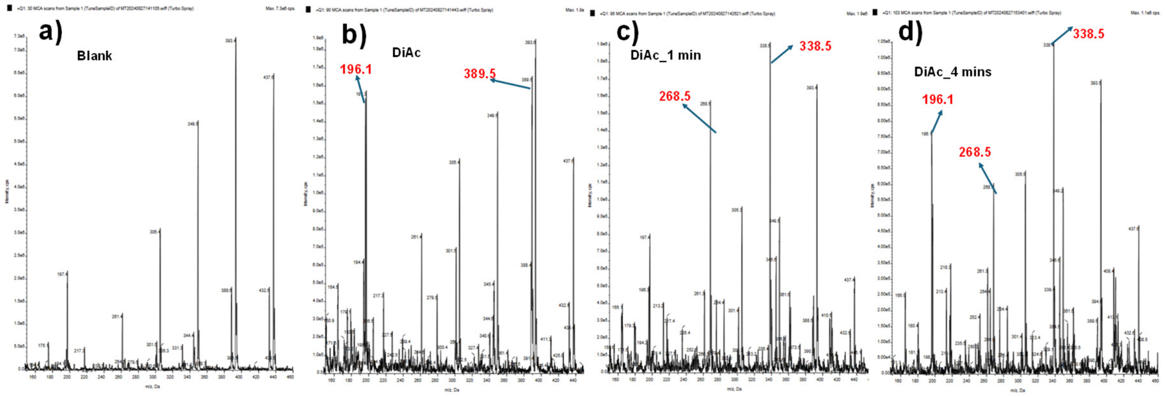

Figure S14. Mass spectroscopy of a) instrument background and b)THF solution of DiAc before UV irradiation, c) after 1-min UV irradiation and d) after 4-min after UV irradiation.

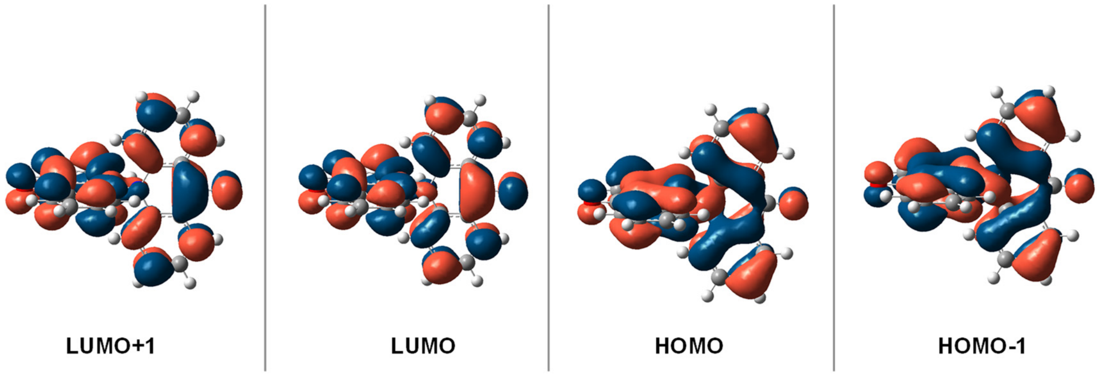

Figure S15. The degeneracy of LUMO/LUMO+1, HOMO/HOMO-1.

Table S3. The energy level and oscillator strength of DiAc at different excited states.

Excited states	Energy level	Oscillator strength
T_1	2.9771 eV	f= 0.00000
T_2	2.9778 eV	f= 0.00000
T_3	3.2437 eV	f= 0.00000
T_4	3.2439 eV	f= 0.00000
T_5	3.2968 eV	f= 0.00000
T_6	3.3430 eV	f= 0.00000
T_7	3.3467 eV	f= 0.00000
S_1	3.7002 eV	f= 0.00000
S_2	3.7005 eV	f= 0.00000
S_3	3.8329 eV	f= 0.00000

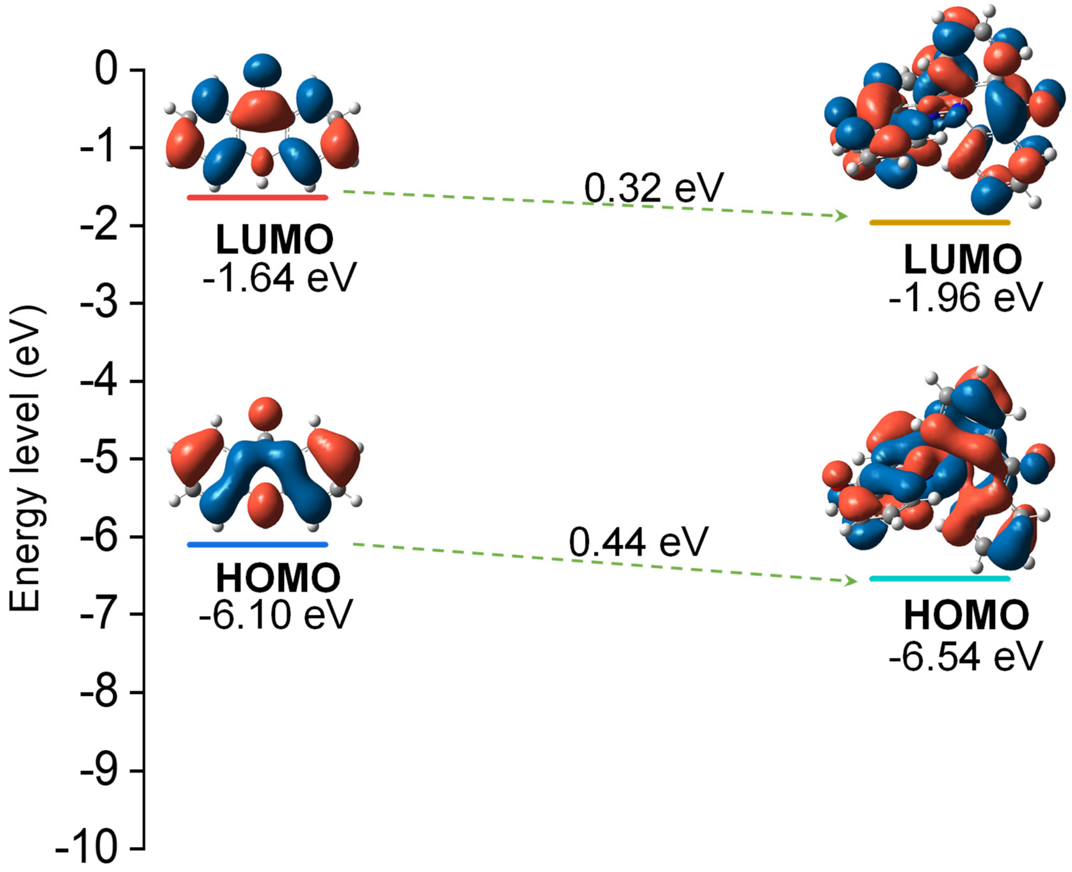

Figure S16. The HOMO and LUMO energy levels of Ac and DiAc.

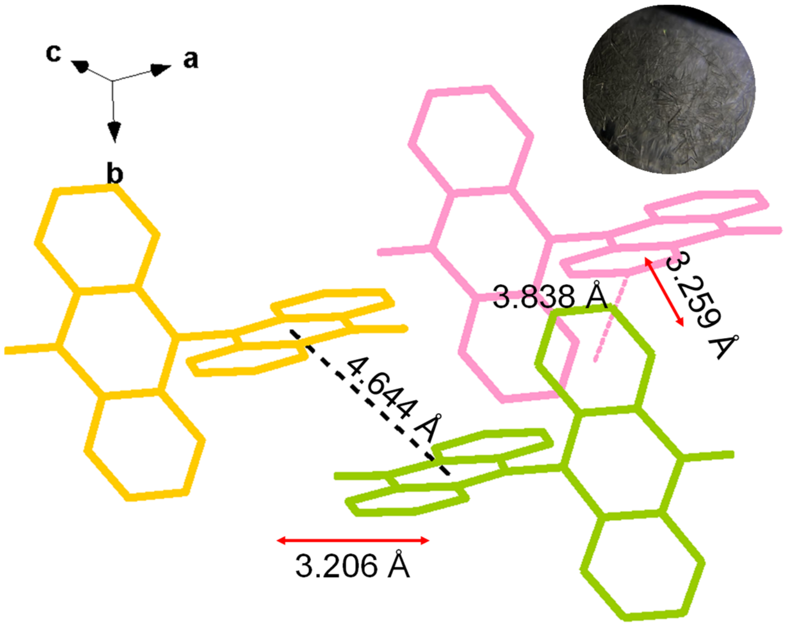

Figure S17. The slippery distances of DiAc in crystals.

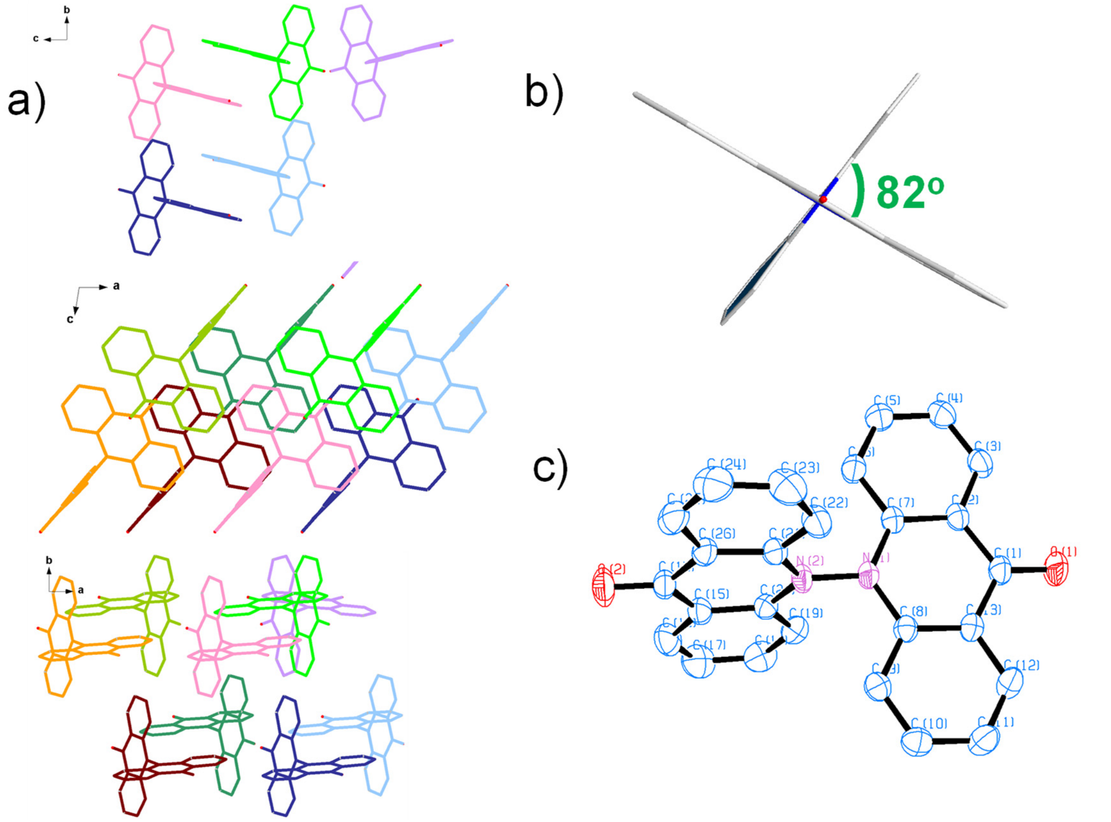

Figure S18. a) The packing mode of DiAc in different direction view, b) The side view of DiAc, and c) The ORTEP diagram of DiAc (40% thermal probability).

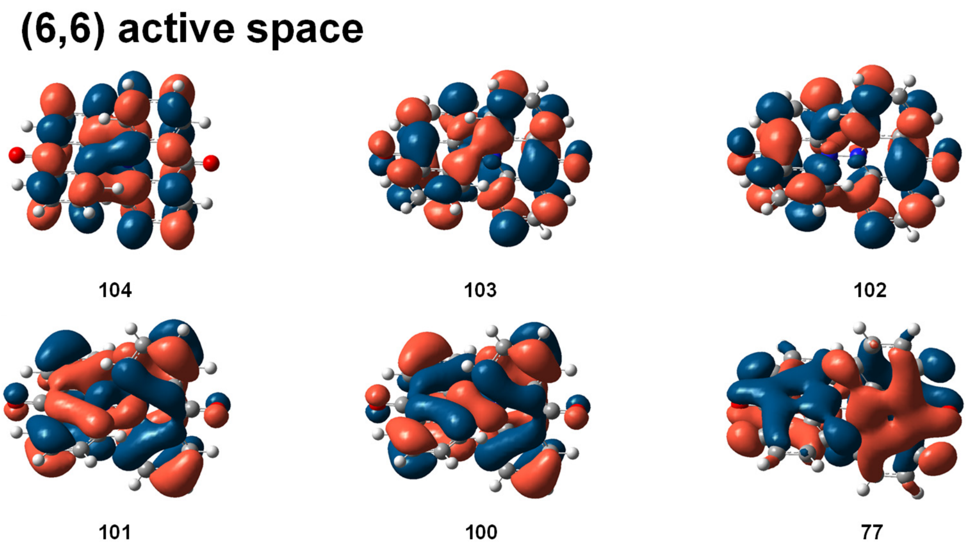

Figure S19. Active MOs for DiAc.

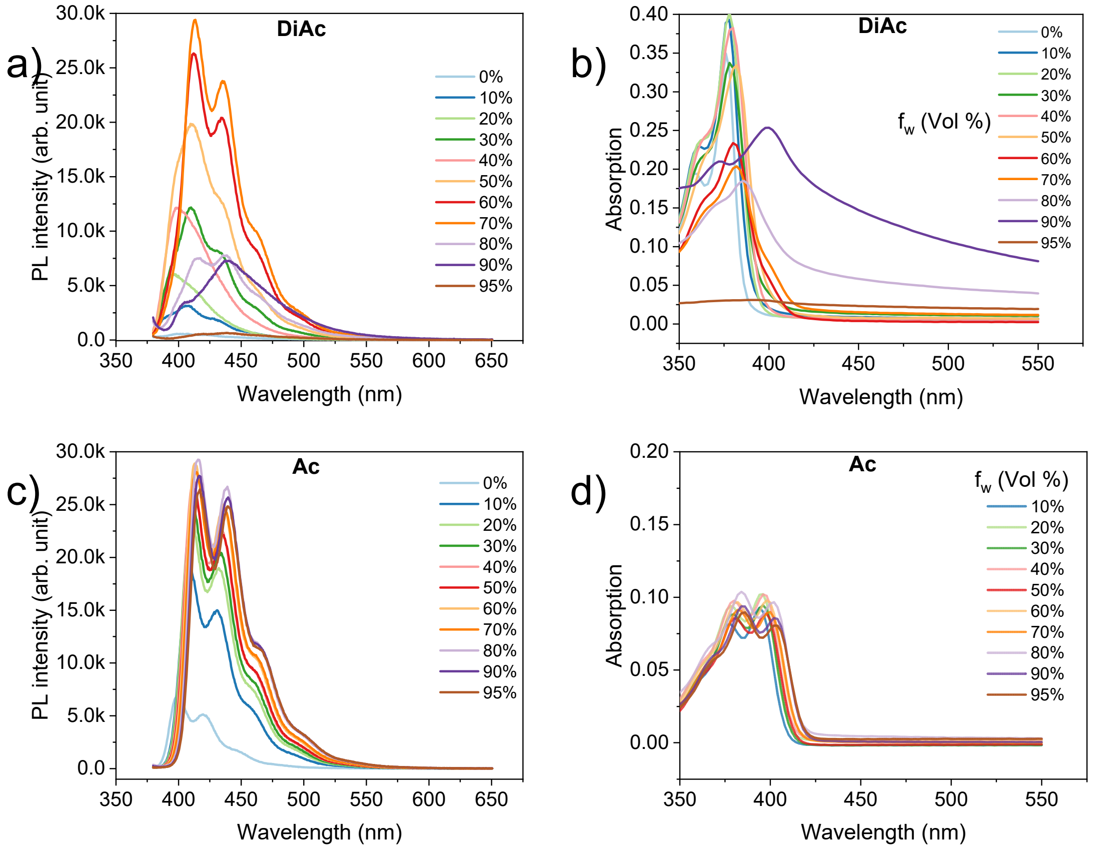

Figure S20. a) PL spectra of DiAc in THF/H_2O mixtures with different water fractions (λ_{ex}=365 nm),b) UV-vis absorption spectra of DiAc in THF/H_2O mixtures with different water fractions, c) PL spectra of Ac in THF/H_2O mixtures with different water fractions (λ_{ex}=365 nm), d) UV-vis absorption spectra of Ac in THF/H_2O mixtures with different water fractions.

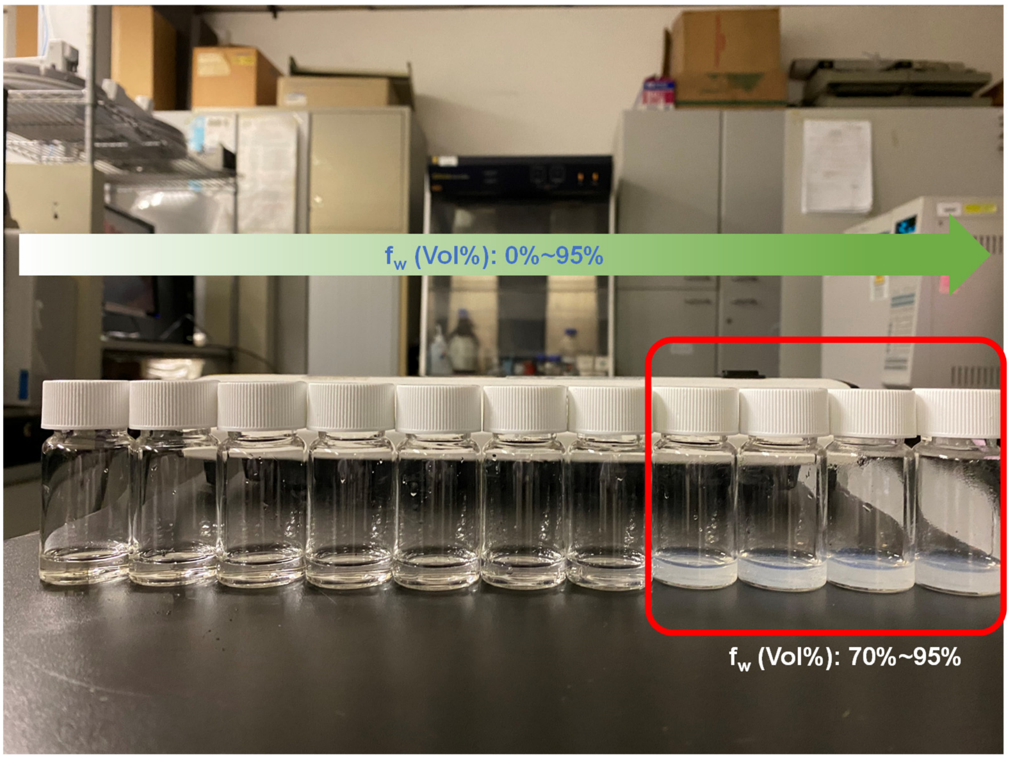

Figure S21. The photography of DiAc in THF/H_2O mixtures with different water fractions.

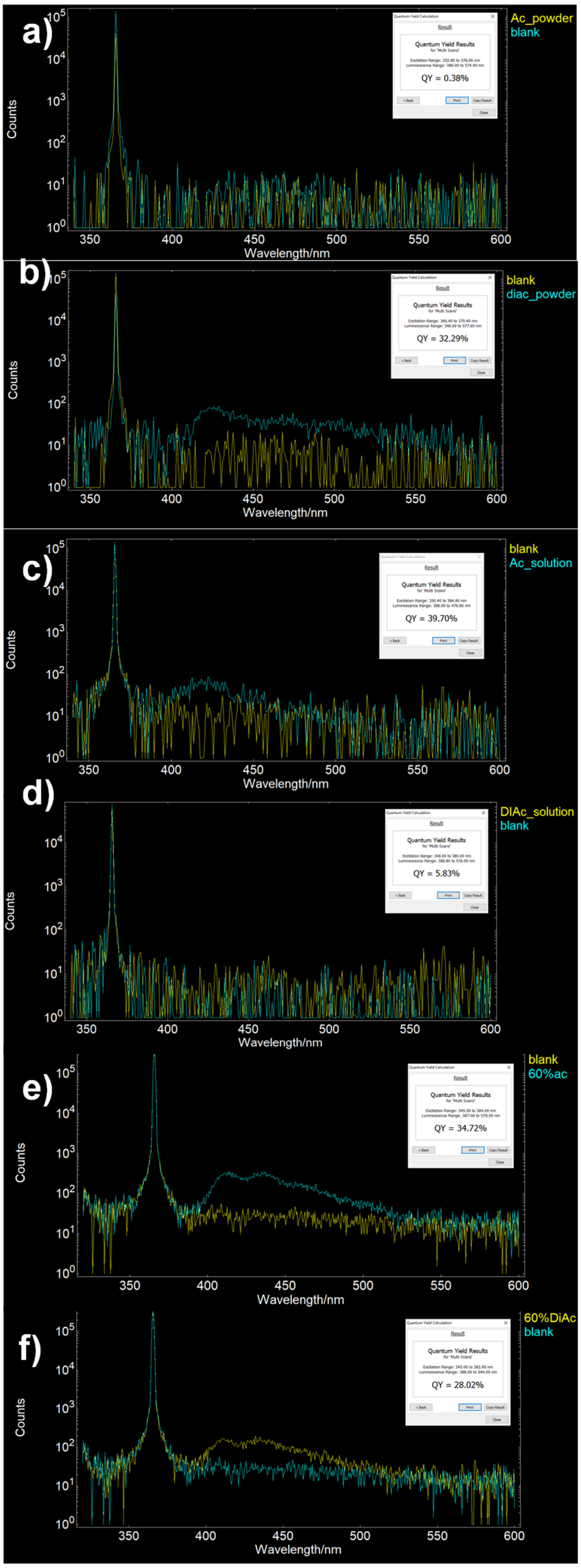

Figure S22. The PLQY (λ_{ex}=365 nm) of Ac and DiAc with a) Ac powder, b) DiAc powder, c) Ac in pure THF solution, d) DiAc in pure THF solution, e) Ac in THF solutions with

60% water, and f) DiAc in THF solutions with 60% water.

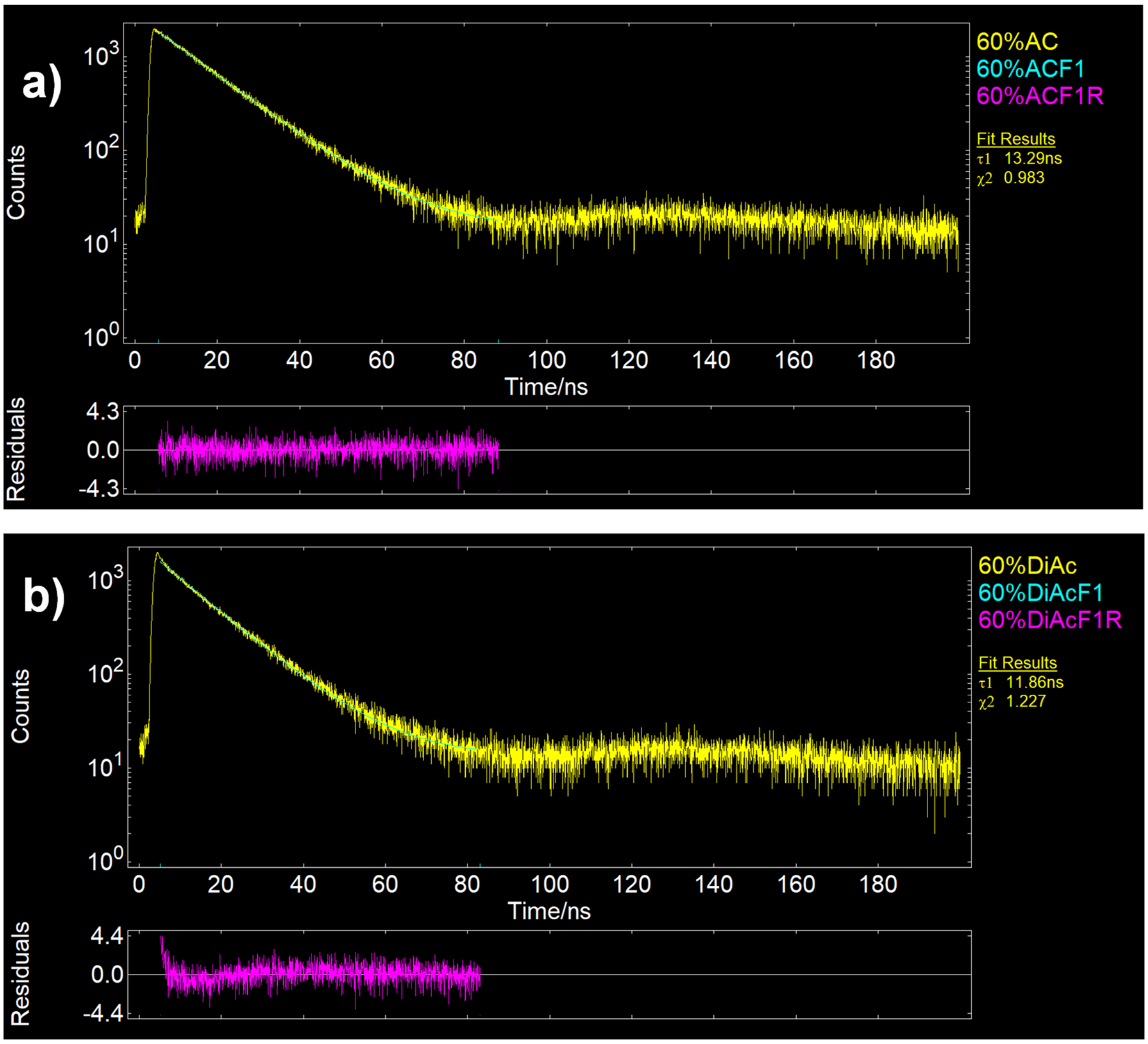

Figure S23. The decay of THF solutions with 60% water fractions(λ_{ex}=375 nm of laser) a) Ac, b) DiAc.

Table S4. The C=O length of DiAc in different states

	C=O
Single crystals	1.2292Å
S_0	1.2311Å
S_1	1.3028Å
